# A randomized, double blind, cross-over, placebo-controlled clinical trial to assess the effects of Candesartan on the insulin sensitivity on non diabetic, non hypertense subjects with dysglyce mia and abdominal obesity. "ARAMIA"

**DOI:** 10.1186/1745-6215-7-28

**Published:** 2006-09-07

**Authors:** Patricio López-Jaramillo, Lina P Pradilla, Vicente Lahera, Federico A Silva Sieger, Christian F Rueda-Clausen, Gustavo A Márquez

**Affiliations:** 1 VILANO Group. Research Institute, Fundación Cardiovascular de Colombia (FCV), Calle 155 A # 23-58, Third Floor, El Bosque sector E, Floridablanca, Santander, Colombia; 2 Research Direction, Medical School, University of Santander, Colombia; 3 Physiology Department – School of Medicine, Universidad Complutense de Madrid. Avenida de la Complutense S/N. 28040 Madrid, Spain

## Abstract

**Background:**

The raising prevalence of type-2 diabetes mellitus and obesity has been recognized as a major problem for public health, affecting both developed and developing countries. Impaired fasting plasma glucose has been previously associated with endothelial dysfunction, higher levels of inflammatory markers and increased risk of developing insulin resistance and cardiovascular events. Besides life-style changes, the blockade of the renin-angiotensin system has been proposed as a useful alternative intervention to improve insulin resistance and decrease the number of new type-2 diabetes cases. The aim of this clinical trial is to study the effect of the treatment with Candesartan, an angiotensin II receptor antagonist, on the insulin resistance, the plasma levels of adipoquines, oxidative stress and prothrombotic markers, in a group of non diabetic, non hypertensive, dysglycemic and obese subjects.

**Methods and design:**

A randomized, double blind, cross-over, placebo-controlled, clinical trial was designed to assess the effects of Candesartan (up to 32 mg/day during 6 months) on the Homeostasis Model Assessment (HOMA) index, lipid profile, protrombotic state, oxidative stress and plasma levels of inflammatory markers. The participants will be recruited in the "Fundación Cardiovascular de Colombia". Subjects who fullfil selection criteria will receive permanent educational, nutritional and exercise support during their participation in the study. After a 15 days-run-in period with placebo and life-style recommendations, the patients who have a treatment compliance equal or greater than 80% will be randomlly assigned to one of the treatment groups. Group A will receive Candesartan during 6 months and placebo during 6 months. Group B will receive placebo during the first 6 months, and then, Candesartan during the last 6 months. Control visits will be programed monthly and all parameters of interest will be evaluated every 6 months.

**Hypothesis:**

Treatment with Candesartan, could improve the HOMA index, the response to the oral glucose tolerance test and reduce the plasma levels of adipoquines, oxidative stress and prothrombotic markers, in non diabetic, non hypertense subjects with dysglycemia and abdominal obesity, recruited from a population at high risk of developing insulin resistance. These effects are independent of the changes in arterial blood pressure. Trial registration: NCT00319202

## Background

During the second half of the 20^th ^century the prevalence of type-2 diabetes mellitus (DM2) has increased dramatically all over the world [1]. It has been estimated that more than 171 million people suffer from this disease and that this number could increase to 366 million by 2030, of which 298 million would be from developing countries [2]. Currently, in Latin America, the DM2 prevalence ranges from 1.2% to 8%, and it is expected to increase to 38% during the next 10 years [3]. This epidemic has been related with the growing prevalence of obesity, especially abdominal [4]. Recently, we have demonstrated that the presence of cardiovascular risk factors in the Andean population is associated with a lower cut-off point of waist circumference [5,6] than the one used for Caucasian populations [7]. We have previously described that Andean population with pathologies like metabolic syndrome, hypertension and preeclampsia, all of them associated with an increased risk of cardiovascular diseases (CVD), presents higher plasma levels of inflammatory markers such as C-reactive protein (CRP) and proinflammatory cytokines [8-11].

Patients with DM2 have an increased risk of developing coronary artery disease by 2 to 4 fold and a higher risk of presenting recurrent and/or mortal cardiovascular events by 1.5 to 3 fold [12]. Based on the risk of developing micro-vascular complications, the American Association of Diabetes (ADA) reduced the threshold for DM2 to a fasting glucose level ≥126 mg/dl [13]. Moreover, the criteria of impaired fasting glucose (IFG) was defined as fasting glucose plasma level between ≥ 100 mg/dL and <126 mg/dL, and was proposed as equivalent to the impaired glucose tolerance (IGT) category obtained by oral glucose tolerance test (OGTT) [13]. Several articles have shown that patients with IFG have an increased risk of cardiovascular morbidity and mortality [14,15]. This association has been explained by the participation of hyperglycemia, especially postprandial, in the development of atherosclerosis due to metabolic and structural changes of the vascular wall [16]. In the long term, this condition may result in macrovascular alterations and in the increased risk of CVD [15]. According to these observations, our group has recently showed that patients with IFG, regardless of other traditional cardiovascular risk factors, presented a higher risk of luminal coronary disease [17].

The etiological factors of DM2 have not been totally elucidated, but it is well known that insulin resistance precedes the DM2 onset by 10 to 20 years [18,19]. Individuals with insulin resistance have impaired endothelium-dependent vasodilation and a loss of the physiological vasodilation caused by insulin [20], which is primarily due to an increased expression of the endothelial nitric oxide synthase (eNOS) [21]. It is also well known that obesity, hypercholesterolemia and diabetes are related to a reduction in flow-mediated dilation (FMD) [22,23], which is mainly caused by the endothelial production of nitric oxide (NO).

Endothelial dysfunction is a condition defined as a decrease in the production of NO and the predominance of vasoconstrictor substances that result in an increase of the the vascular tone that predisposes to a prothrombotic and proatherogenic state [24,25].

**Figure 1:**
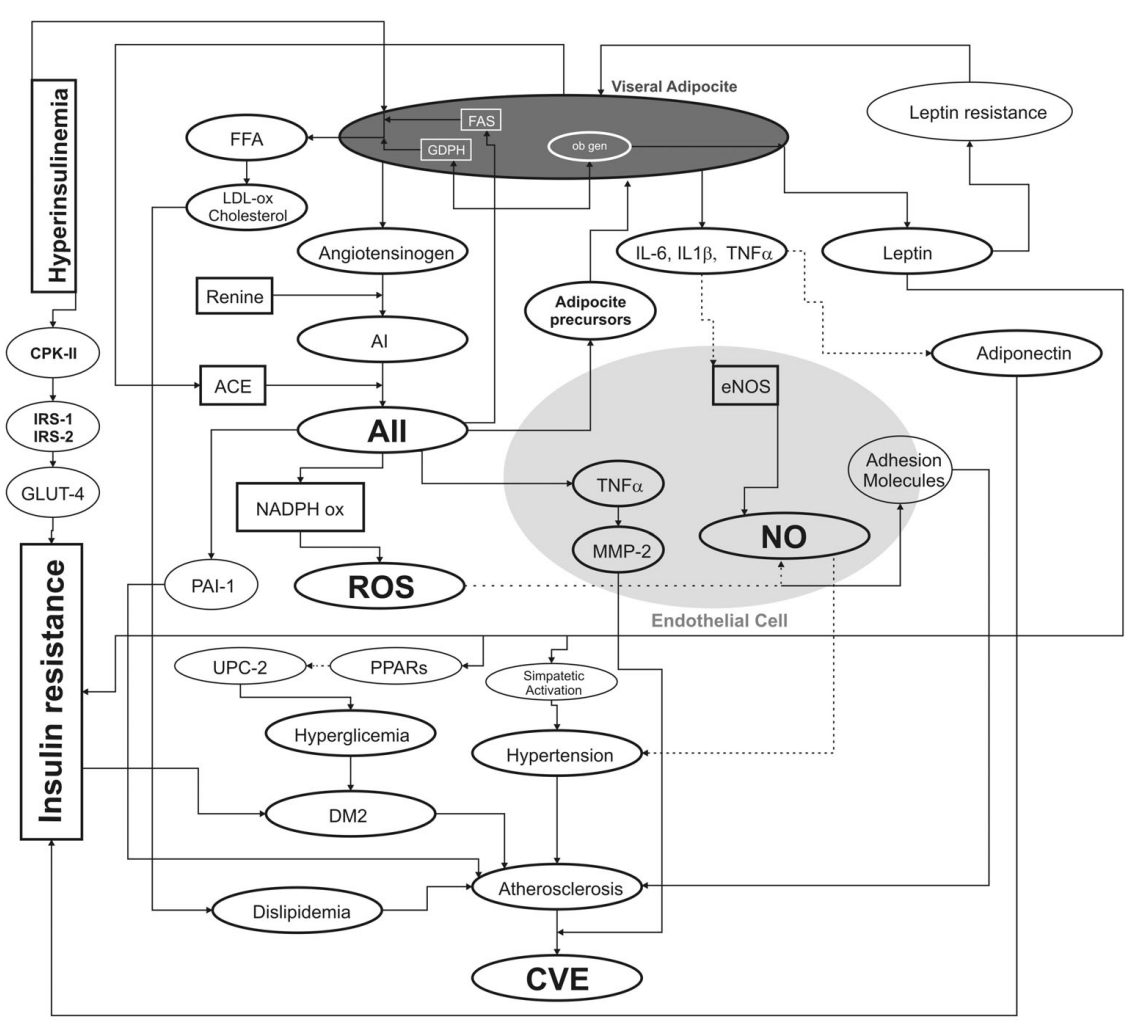
**title**. Physiopathological mechanisms relating abdominal adiposity, angiotensin II, insulin resistance and cardiovascular events. For description see text.**Foot note.FFA: **Free Fatty Acids, **FAS:**, **GDPH:**, **IL-6: **Interleukine 6, **IL1β: **Interleukine 1β, **TNFα: **Tumoral Necrosis Factor α, **AI: **Angiotensin I, **AII: **Angiotensin II, **ACE**: Angiotensin Converting Enzime, **eNOS**: endothelial Nitric Oxide Sintase, **CPKII: **Creatinphosphokinase-II, **IRS-1: **Type 1 Insulin Soluble Receptor **IRS-2: **Tipe 2 Insulin Soluble Receptor, **GLUT-4:**Type 4 Glucose Membrane Trasnporter, **MMP2: **Matrix Metalloproteinase-2, **NO: **Nitric Oxide, **UPC-2: **Uncoupling Portein 2, **PPARs: **Peroxisome Proliferator-Activated Receptors, **DM2: **Tipe 2 Diabetes Mellitus, **CVE: **Cardiovascular Events, scater line: Inhibition or delay, filled line: Activation or increment.

Intra-abdominal adipocytes are directly related to hyperinsulinemia and insulin resistance [26]. Hyperinsulinemia promotes the release of free fatty acids (FFA) from the adipocytes and their hepatic transformation into oxidized LDL, which has a higher atherogenic potential. Moreover, abdominal adipocytes are an important source of proinflammatory cytokines, such as tumor necrosis factor alpha (TNF-α) and interleukin 6 (IL-6) [27], that can reduce the expression and activity of the eNOS in human cultivated umbilical endothelium cells [28] which suggests that this could be one of the mechanisms by which abdominal obesity is related to endothelial dysfunction [29]. Furthermore, we have recently demonstrated that in cultured human endothelial cells, angiotensin II (AII), through its type 1 receptor (AT_1_), stimulates the TNF-α production which increases the activity of matrix metalloproteinase-2 (MMP-2)[30], enzyme that affects the endothelium structure as well as the stability of the atherosclerotic plaque [31] (Figure 1).

All of them, angiotensin receptors (AT_1 _and AT_2 _types), angiotensin converting enzyme (ACE) and angiotensinogen, are widely expressed in the human adipose tissue [32,33]. Furthermore, the angiotensinogen gene expression is directly related to the degree of abdominal obesity [34]. Moreover, a direct correlation between body mass index and the circulating levels of angiotensinogen and AII has been reported [35]. These results demonstrate that the visceral adipocyte is both, a generator and a target tissue of the AII through the activation of AT_1 _and AT_2 _receptors. AII has regulatory functions in the adipogenesis and the size of the fat reservoir. Experiments *in vitro *have shown that AII indirectly induces the transformation of adipocytes precursor cells into mature fat cells [36], suggesting that this is one of the mechanisms by which AII stimulates the accumulation of the adipose tissue. In studies realized in rodents, the treatment with AII Receptor Blockers (ARBs), caused a reduction in the weight and in the size of the adipocytes independently of the food intake [37]. It is normally accepted that AT_2 _receptors are the most commonly and widely distributed receptors of AII, however, effects of AII mediated by AT_1 _receptors on the storage of triglycerides and on the activity of key lipogenic enzymes such as fatty acid synthase (FAS) and glycerol-2 dehydrogenate phosphate (GDPH) have also been demonstrated in cultured cells [38].

The increase in the production and storage of FFA induces insulin resistance through the inhibition of the transportation and phosphorylation of glucose in skeletal muscle, which produces a reduction of the glycogen synthesis and glucose oxidation rates [39]. Additionally, it has been suggested that FFA interfere with the stimulation of the expression of the glucose transporter GLUT-4 and the hexokinase activity [39]. AII and insulin share signal transduction pathways. Thus, insulin activates proteinkinase C (PKC) through the tyrosine phosphorilation of insulin receptor substrate type 1 and 2 (IRS-1 and IRS-2) and stimulates the MAP-kinase pathway signaling, whereas the AII inhibits PKC that alters the intracellular signaling of insulin [40].

All also has a stimulating effect on the transcription rate of the *ob *gene in human adipocytes, which codifies leptin, inhibiting appetite and regulating thermogenesis [41]. The sustained increase in leptin levels leads to leptin-resistance, moreover, high levels of leptin produce a greater expression of the uncoupling protein 2 (UPC-2) mediated by peroxisome proliferator-activated receptors (PPARs). UPC-2 interferes with the mitochondrial respiratory chain of the pancreatic β-cells, reducing the ATP generation and blocking the first peak of insulin secretion and favoring the onset of hyperglycemia [42]. Contrary to the results obtained in *in vitro *experiments [43], the infusion of AII to animals causes no effect over leptine expression [44]. This contradictory result could be explained by the activation of the sympathetic nervous system, which impedes the stimulating effect of AII on leptin production. Furthermore, high concentrations of leptin increase blood pressure, through sympathetic nervous activation and the sodium retention in the kidneys contributing to the development of hypertension in obese subjects [45]. The administration of ARBs in animal models, increase adiponectin plasma levels [46], which stimulate glucose utilisation and fatty-acid oxidation by activation of AMP-kinase [47]. In fact, the administration of Candesartan, an antagonist of AT_1 _receptor, to a group of hypertense subjects caused a reduction of adiponectin, C-reactive protein and markers of insulin resistance, such as the QUICKI index [48]. Furthermore, TNFα, another proinflammmatory cytokine, down-regulates the expression of adiponectin gene, impairs signal transduction of insulin in muscles [49] and it is proposed as a factor involved in the etiology of obesity related insulin resistance syndrome.

All acts also as a pro-oxidant substance by regulating the activity of the enzyme NADPH oxidase that catalyzes the production of reactive oxygen species (ROS), such as the superoxide anion and peroxynitrite[50]. ROS are substances that are directly related to the development of atherosclerotic plaque not only by inactivating NO but also by increasing the expression of cellular adhesion molecules that favor the migration of monocytes and leukocytes to the vascular wall [51] and by stimulating the growth, the remodeling and the migration of smooth muscle cells [52]. Moreover, AII has a procoagulant effect by stimulating the production of Plasminogen Activator Inhibitor-1 (PAI-1) [53], which is the principal endogenous inhibitor of fibrinolysis "in vivo" and is associated with atherosclerosis [54].

The treatment with ACE inhibitors (ACEIs) and ARBs has been shown to improve the peripheral insulin resistance in both, animal [55] and clinical models [56]. The mechanism by which the renin-angiotensin-aldosterone system (RAS) blockade has a beneficial effect on the responsiveness to insulin has not been totally clarified. Some changes in peripheral insulin sensitivity after treatment with ACEIs or ARBs might be partially mediated by changes in local blood flow to sites of glucose uptake [57]. However, a variety of non-haemodinamic effects have also been reported. In obese Zucker-type rats, it was demonstrated that the chronic administration of a selective ARBs produced a significant increase in the GLUT-4 expression in skeletal muscle, a reduction in the concentrations of plasma fatty acids and an improvement in the responsiveness to insulin [58]. Moreover AII also increases the expression of Hexokinase, a key enzyme in glucose metabolism of skeletal muscle [59].

ARBs have also been associated with an increase of the PPARγ activity [60] which plays an important role in the regulation of insulin action, by controlling the transcription of multiple genes involved in the metabolism of lipids and glucose [61]. Even in absence of AT_1 _receptors, ARBs were shown to promote the activation of PPARγ in human adipose cells, which supports the existence of additional mechanisms by which ARBs improve the responsiveness to insulin [61,62].

Some clinical trials have demonstrated that ACEI and ARBs reduce the number of new-onsets DM2 when compared with other anti-hypertensive therapies [63,64]. In the study SCOPE [65] the use of Candesartan as an antihipertensive treatment, was associated with a relative reduction in new-onset diabetes by 19% (95% IC- 2 to 42%, p = 0.09) during a mean follow-up period of 3.7 years. This result is consistent with observations in other studies using blockers of the RAS in subjects with hypertension [66] including the LIFE study that compared Losartan and atenolol [67], the ALPINE study that compared Candesartan and hydroclorotiazide HCT [68], and the VALUE study that compared Amlodipine and Valsartan[63]. Moreover, results from studies conducted in normotense subjects, like the CHARM study [69], in which ARBs, caused a reduction in new-onsets DM2, suggest that the observed effects of these medications over the development of DM2 are independent of their effects over blood pressure. However, to this day, we lack results from clinical trials aimed specifically at describing the effects of the ARBs over dysglicemia/IGT in normotense non diabetic and obese subjects.

Although the mechanisms are still speculative, the results discussed above suggest that AII produced in the adipocytes of subjects with abdominal obesity is associated with insulin resistance syndrome and dysglycemia, and support the conduction of clinical trials oriented to demonstrate the beneficial effect of ARBs in the insulin sensitivity of individuals at high risk of developing CVD and DM2.

## Hypothesis

Candesartan (32 mg/day during 6 months) has a beneficial effect on fasting plasma glucose, proinflammmatory, prothrombotic and oxidative stress markers in non diabetic, non hypertense subjects, with dysglicemia and abdominal obesity. The improvement of these markers is independent of changes in arterial blood pressure.

## Methods/design

### General objective

To evaluate the effect of Candesartan 32 mg/d during 6 months on the sensibility to insulin, the concentration of inflammatory adipoquines and the prothrombotic and oxidative stress markers in non diabetic, non hypertense subjects with dysglycemia and abdominal obesity, recruited from a population with high risk of developing metabolic syndrome and insulin resistance.

#### Specific objectives

▪ To establish the effect of the administration of Candesartan on the insulin resistance assessed through the HOMA index, fasting plasma glucose, OGTT, and HbA1c levels.

▪ To study the effect of Candesartan 32 mg/d administrated during 6 months on fasting plasma levels of adipoquines such as leptin, resistin, adiponectin, IL-6 and CRP.

▪ To evaluate the effect of Candesartan on prothrombotic markers, such as D dimer, tPA/PAI-1 ratio.

▪ To determine whether the administration of Candesartan decreases the concentration of oxidative stress markers such as plasma oxidized/reduced glutathione ratio, total oxidative capability, malonaldehyde and urinary 8-Isoprostanes.

▪ To determine whether the effects of Candesartan on the insulin sensibility, adipoquines and oxidative stress, are independent of its effect upon the blood pressure.

#### Study design

**Figure 2:**
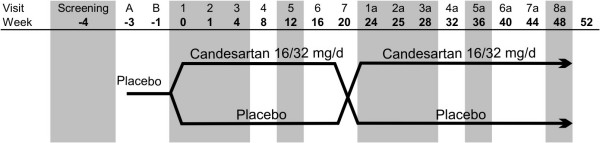
**title**. Study design.

Randomized, double blind, placebo-controlled, cross-over clinical trial (Figure 2).

#### Study treatments

▪ **Treatment A: **Candesartan 16 mg (one tablet per day) taken with breakfast during 4 weeks and then increased to the optimal dose of 32 mg/d (two tablets) during the next 20 weeks, depending on the subjects' tolerance.

▪ **Treatment B: **Placebo tablets administered similarly to treatment A, one tablet daily during 4 weeks and then 2 tablets per day during the next 20 weeks.

All the subjects will be included in a therapeutic life-style change program (TLC: educational, nutritional and exercise support) during the study.

#### Study groups

The study embraces two arms (Figure 2):

▪ **Group 1: **will receive the treatment A during the first 24 weeks and then the treatment B during the last 24 weeks.

▪ **Group 2: **will receive the treatment B during the first 24 weeks and then the treatment A during the last 24 weeks.

#### Population

Participants will be non diabetic non hypertense individuals of both genders, older than 18 years with abdominal obesity and dysglycemia and/or IGT. Inclusion and exclusion criteria are shown in Table 1

**Table 1:** Selection criteria

**Inclusion criteria **
• Men and women older than 18 years of age.
• Waist circumference ≥ 90 cm in male and ≥ 80 cm in female.
• To have fasting plasma glucose between 100 and 125 mg/dL and/or OGTT at 2 hours ≥ 140 mg/dL and <200 mg mg/dL.
• Having a treatment compliance over 80% at the end of the run-in phase.
All women with bearing potential must have a secure contraceptive method. Secure method will be considered: surgical sterilization, postmenopause condition with an age greater than 45 years and a period of amenorrhea ≥ 2 years. (In premenopausal women, the use of hormonal method or two barrier contraceptive methods including 1 month after the conclusion of the active phase of study treatment).
**Exclusion criteria**
• Prior diagnosis of type 1 or 2 diabetes mellitus, chronic or acute renal insufficiency, coronary disease clinically evident (acute myocardial infarction, chest angina, myocardial revascularization) or cardiac insufficiency, or history of prior cardiovascular events (AMI, CVD, or CABG).
• Significant chronic disease (terminal stage cirrhosis or hepatic disease or cancer) that affects the survival of patients at 24 months.
• Chronic inflammatory diseases except obesity (lupus, rheumatoid arthritis, etc.).
• Blood arterial pressure >140/90.
• Acute infection of any etiology within 4 weeks prior to the beginning of the study.
• Use of steroid hormones or NSAIDs 4 weeks prior to the beginning of the study.
• Patient enrolled in a program or treatment to lose weight, 8 weeks prior to the study entry.
• Patient requiring treatment with immunosuppressive agents, for any circumstance.
• Patient who has participated in a clinical trial in the 8 weeks prior to the study entry.
• Patient who requires a major surgical procedure during the next 12 months, after enrollment (Abdominal or thoracic surgery, vascular, neurosurgery, urologic or gynecologic surgical procedure).
• Patient with history of severe chronic gastritis or any condition of the gastrointestinal tract that may affect the absorption and/or distribution of any drug administered orally.
• Alteration of the hepatic function tests.
• Triglycerides ≥ 600 mg%.
• Serum creatinin ≥ 1.5 mg/dl or calculated creatinin clearance (Cockroft's method) less than 50 ml/min.
• History of use of psychoactive drugs or abuse of alcohol.
• Positive pregnancy test in the screening visit.
• Concomitant treatment with any other antihypertensive drug.
• Contraindication to receive treatment with Candesartan.
• Pathological alterations of aortic or mitral cardiac valves (stenosis o insufficiency) or hypertrophy cardiomyopathy.
• Denial to sign informed consent, or any mental condition that makes the patient part of a susceptible population

#### Sample size

A sample size of 84 subjects was estimated, considering a crossover clinical trial design as proposed by Hills and Armitage [70], accepting a type I error of 0.05, a power of 90%, and assuming a difference of 20 % in the HOMA index after 6 months of treatment with Candesartan (3 to 2.4) and a maximum standard deviation of 1.5. The final size of the sample, adjusted for a drop-out of 8%, is 100 subjects (50 in each group). The sample size ensures a power of 90% to detect differences in fasting glycemia of at least 8 mg/dL (0.44 mMol/L) with a standard deviation (SD) of 20 mg/dL (1.1 mMol/L), or a difference of 14 mg/dL (0.77 mMol/L) in the 2 hours post load glycemia with a SD of 40 mg/dl (2.2 mMol/L).

### Procedures

#### Enrollment

This study will be realized in an adult population with a maximum enrollment period of one year. Screening visit will include a semi-structured interview, anthropometry and blood pressure evaluation. Eligible subjects will be scheduled one week later for "Visit A", to perform a new interview, a physical examination and to withdraw blood samples, after a 10 hour fasting period, to determine plasma glucose levels, lipid profile, hepatic and kidney function and OGTT. Those who fulfill screening criteria will be included in a run-in phase to receive placebo and the standard treatment with TLC. The patients will be blinded during this phase, which will last 2 weeks. The patients with a compliance equal or greater than 80% during this "Run in" phase will be included in the study.

### Baseline assessments

Visit B will include measurements of blood pressure, anthropometric parameters, OGTT and electrocardiogram. A fasting blood sample and a 24 hour urine sample will be taken and stored (-70°C) to determine glucose, HbA1c, insulin, IL-6, leptin, resistin, adiponectin, tisular plasminogen activator (tPA), PAI-1, oxidized/reduced glutathione, malonaldehyde and 8-isoprostanes in urine. Once the patient completes the foregoing steps, during visit 1 he/she will be randomized to one of the arms of the treatment. For this purpose, a randomization system by blocks of 4 will be used. New tests will be performed at the end of each treatment, according to the study flowchart (Table 2). Randomization and preparation of medication/placebo will be done by AstraZeneca at Mölndal, Sweden.

**Table 2:** Study flowchart.

**Visit**	**Screening**	**A**	**B**	**1**	**2**	**3**	**4**	**5**	**6**	**7**	**1a**	**2a**	**3a**	**4a**	**5a**	**6a**	**7a**	**8a**	
**Week**	**-4**	**-3**	**-1**	**0**	**1**	**4**	**8**	**12**	**16**	**20**	**24**	**25**	**28**	**32**	**36**	**40**	**44**	**48**	**52**
**Passive Follow up**																			x
**Nurse interview**	x	x	x	x	x	x	x	x	x	x	x	x	x	x	x	x	x	x	
**MD interview**		x	x	x	x	x		x			x	x	x		x			x	
**Medication delivery**		x		x		x	x	x	x	x	x		x	x	x	x	x		
**Physical examination**	x			x	x	x		x			x	x	x		x			x	
**Randomization**				x															

**Sample withdrawing**		x	x			x		x			x		x					x	
**Sample storage**			x								x							x	
**ECG**			x																
**Hemogram**			x								x							x	
**Plasma glycemia**						x		x					x		x				
**Lipid Profile**		x									x							x	
**Hepatic Function test**		x									x							x	
**Renal Function test**		x									x							x	

**OGTT**		x									x							x	
**Insulin**			x								x							x	
**CRP**				x							x							x	
**IL-6**				x							x							x	
**Hb A1c**				x							x							x	
**Leptin**				x							x							x	
**Resistin**				x							x							x	
**Adiponectin**				x							x							x	

**TPA**				x							x							x	
**PAI-1**				x							x							x	

**8-Isoprostanes**				x							x							x	
**Malonaldehyde**				x							x							x	
**Oxi/Red Glutathione**				x							x							x	

### Active follow-up

Seven days after beginning the treatment, and every month thereafter, the subjects will be asked to return for a visit in order to verify compliance, evolution of blood pressure and occurrence of adverse events. In the months 1 and 3 of each treatment, new fasting glucose determination will be done in all subjects. All basal measurements will be repeated at the end of each treatment (every 6 months).

### Passive follow-up

All the subjects will undergo a passive follow-up (telephonic follow-up) 30 days after concluding the treatment.

### Blood samples

In fasting conditions (at least 10 hours), blood samples will be taken from the antecubital vein, with appropriate conditions of asepsia and antisepsia, using 3 vacutainer tubes, one dry, another with citrate, and the other containing EDTA. After 10 minutes in vertical position, all samples will be centrifugated at 3000 rpm during 15 minutes to extract the serum or plasma. Part of the samples obtained during visits B, 5 and 9 will be stored in Ependorf vials at -70°C until the end of the study.

#### Anthropometrical measurements

All anthropometrical measurements will be taken first thing in the morning after urine elimination, with the subject using light clothing and no shoes.

**Weight**: will be measured with the patient standing and then registered after rounding it to the nearest 200 grams. The weight scale will be calibrated to 0 before each measurement.

**Height**: will be measured using a metric tape with the patient standing against the wall in Frankfort's position, and the value marked by a ruler placed horizontally on the head of the patient.

**Heart rate**: number of beats per minute will be measured in the radial artery.

**Blood pressure**: will be taken twice (with a difference of 5 minutes between the measurements) using a mercury sphygmomanometer in 2 occasions on the right arm, with the patient comfortably seated, after a 5 minute rest. Systolic blood pressure (SBP) will be determined by the first audible sound (Korotkoff phase 1). Diastolic blood pressure (DBP) will be registered when the sound disappears (Korotkoff phase 5). The patient should not have smoked 30 minutes prior to the blood pressure measurement. The pneumatic arm cuff must cover 2/3 of the upperarm's length; its inferior border must be 2–3 cm over the antecubital space; the cuff will be slowly deflated. The mean blood pressure (MBP), will be calculated using the following formula [SBP+(2*DBP)]/3

**Waist circumference**: will be measured in 2 occasions with the patient in a standing position, with the arms on the sides and using a measuring tape adhered to a dynamometer that exerts a force of 750 gr. The measuring tape will be placed horizontally in a middle point between the iliac crest and the anterior costal border. The difference between the two measurements should not be more than 0.5 cm.

**Hip circumference**: will be measured in 2 occasions with the patient in a standing position with the arms on the sides of the body, using a measuring tape adhered to a dynamometer that exerts a force of 750 gr. The hip circumference will be assessed over the major trochanters. The difference between the two measurements should not be more than 0.5 cm.

**Waist-Hip Relation (W/H-R): **will be obtained from the ratio between the waist and hip circumferences.

**Antero-posterior diameter**: will be measured twice with the patient in a decubitus supine position, using a ruler perpendicular to the bed and registering the cutting point with the tape applied horizontally on the abdomen. The difference between the two measurements should not be more than 0.5 cm.

**Body Mass Index (BMI)**: This index will be estimated using the weight in kilograms divided by the second power of the height expressed in meters.

#### Biochemical markers

Routine clinical test and inflammation markers will be processed in the Clinical Research Laboratory from the "Fundación Cardiovascular de Colombia" (Floridablanca, Colombia). The measurement of oxidative stress markers will be processed in the laboratories of Complutense University (Madrid, Spain).

**Glycemia, Lipid profile, Serum Creatinine, Hepatic enzymes (AST/ALT) **will be quantified by a routine colorimetric method. (Biosystems BTS-303 Photometric, España).

**Glycosylated Hemoglobin A1c: **Will be determined with a quantitative automated technique GlycoHemoglobin Analyzer (DCA 2000+ Bayer^®^.) using a whole blood sample.

**Insulin, High-sensitivity C-Reactive Protein and Interleukin 6: **will be determined by high sensitivity chemoluminescent inmunoassay technique (IMMULITE ^® ^Automated Analyzer, Diagnostic Products Corporation, Los Angeles, USA).

**Leukocyte count and differential formula: **will be determined by an automated counter (Baker System 9120 AX ^®^, Biochem Inmunosystem, USA).

**Glucose Tolerance Test: **will be done after a fasting period of at least 10 hours. After urine elimination, an intravenous catheter will be placed in the antecubital vein and blood samples will be withdrawn to assess the baseline blood glucose, then, a glucose load equivalent to 75 gr diluted in 300 mL of water will be administered to each study subject within a period of no more than 10 minutes. Then, after 2 hours, a new blood sample will be withdrawn to assess glycemia. Patients should not eat anything or do any exercise during the test.

**Serum Leptin, resistin and adiponectin: **will be measured by ELISA technique.

**HOMA Index**: Will be obtained from a mathematical model using the following formula:

HOMA=(Glycemia(mg/dL)18.02)∗Insulinemia(mU/mL)22.517
 MathType@MTEF@5@5@+=feaafiart1ev1aaatCvAUfKttLearuWrP9MDH5MBPbIqV92AaeXatLxBI9gBaebbnrfifHhDYfgasaacH8akY=wiFfYdH8Gipec8Eeeu0xXdbba9frFj0=OqFfea0dXdd9vqai=hGuQ8kuc9pgc9s8qqaq=dirpe0xb9q8qiLsFr0=vr0=vr0dc8meaabaqaciaacaGaaeqabaqabeGadaaakeaacqWGibascqWGpbWtcqWGnbqtcqWGbbqqcqGH9aqpdaWcaaqaamaabmGabaWaaSaaaeaacqWGhbWrcqWGSbaBcqWG5bqEcqWGJbWycqWGLbqzcqWGTbqBcqWGPbqAcqWGHbqycqGGOaakcqWGTbqBcqWGNbWzcqGGVaWlcqWGKbazcqWGmbatcqGGPaqkaeaacqaIXaqmcqaI4aaocqGGUaGlcqaIWaamcqaIYaGmaaaacaGLOaGaayzkaaGaey4fIOIaemysaKKaemOBa4Maem4CamNaemyDauNaemiBaWMaemyAaKMaemOBa4MaemyzauMaemyBa0MaemyAaKMaemyyaeMaeiikaGIaemyBa0MaemyvauLaei4la8IaemyBa0MaemitaWKaeiykaKcabaGaeGOmaiJaeGOmaiJaeiOla4IaeGynauJaeGymaeJaeG4naCdaaaaa@680B@

### Quality assurance systems

The inter- and intra-assay variation coefficient will be determined for all measurements. In order to eliminate the inter-assay error, all biochemical determinations will be performed at once.

### Data processing and quality assurance

All study data will be collected by trained personnel. These data will be recorded on forms previously designed for such purpose (Case Report Form-CRF). After completing the CRF, a monitor will review them to assure that they are correctly filled and legible. All corrections will be done by the monitor according to the Good Clinical Practice guidelines. Then, the information will be typed and stored twice in independent data base. A computer program (Epi-Info 2000) will be used to compare both database. Every discrepancy will be printed and corrected using the original CRF as reference.

### Data monitoring

The study coordinator will make sure that data is adequately collected. He/she will register the study visits and the time of data collection and the different procedures, as well as the compliance to the treatment.

### Data management

The FCV Research Institute will be responsible for the data management. Once the data is correctly recorded, the means and ranges will be estimated and the relevant variables will be crossed to identify inconsistencies or extreme values which could result from errors in data management (internal consistency analysis). Any detected error will be corrected using the original form and the lab reports as references, maintaining the 2 original database untouched.

Modifications and statistical procedures during the data analysis will be documented in the Stata 9.0 program, which will allow the replication of the data analysis whenever necessary.

### Statistical analysis

The study is set forth as an efficacy study of Candesartan in the improvement of the insulin sensibility and the OGTT results. The averages and proportions with their corresponding 95% confidence intervals will be obtained in a descriptive analysis for all clinically relevant variables measured during the baseline evaluation. In order to evaluate the presence of differences between the groups, the Student's paried *t- *test, the Wilcoxon's signed-rank test or the McNemar's test will be used according to the variable's characteristics. Linear multiple regression will be used with the purpose of comparing the results of the treatments. The analysis will be performed by the intention-to-treat approach. A p value under 0.05 will be considered as statistically significant.

The primary endpoint for the analysis will be the change in the value of HOMA index, fasting glucose and post-charge glucose plasma levels. The secondary endpoint for the analysis will include the changes in serum insulin, leptin, adiponectin, resistin, CRP, IL-6, tPA/PAI-1 ratio, Oxidized/Reduced glutathione ratio, malonaldehyde and 8-isoprostanes.

Treatment safety will be evaluated by the clinical history review and the statistics of the reported adverse events.

### Safety committee and events assignation committee

A safety and events assignation committee will be created, according to the Harmonized Tripartite Guidelines of the International Conference of Harmonization for Good Clinical Practice.

### Ethical aspects

The clinical trial will be conducted according to the Helsinki's Declaration, the Good Clinical Practice Guidelines and the Colombian legislation (Resolution 8430/93 of the Ministery of Health). The patient will provide written informed consent in a form designed for such purpose. The information generated by the study will be confidential and strictly limited to the purposes stipulated in the protocol. The patient may refuse to continue participating in the study at any moment after providing his/her consent. The study has been approved by FVC ethics committee. All assessments will be performed by trained staff. The blood samples will be collected in aseptic conditions by an expert bacteriologist.

### Study timeline

**Figure 3:**
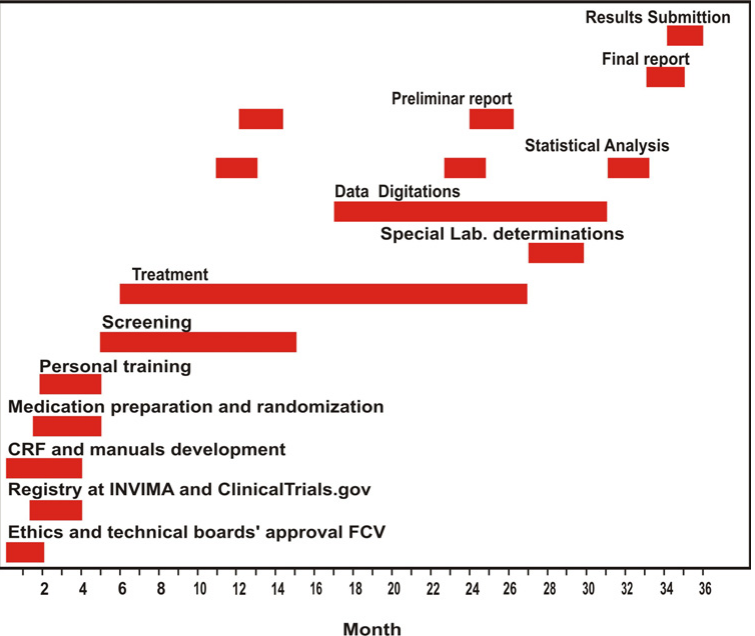
**title**. Study Timeline.

The study will last 36 months. The initiation will be defined by the financial approval from the sponsor(Figure 3.).

## Abbrebiations

**(ACE) **Angiotensin Converting Enzyme

**(ACEIs) **Angiotensin Converting Enzyme Inhibitors

**(ADA) **American Association of Diabetes

**(AII)**Angiotensin II

**(AT_1_) **Angiotensin II Type 1 Receptor

**(AT_2_) **Angiotensin II Type 2 Receptor

**(BMI) **Body Mass Index

**(CRF) **Case Report Form

**(CRP) **C-reactive Protein

**(CVD) **Cardiovascular Diseases

**(DBP) **Diastolic Blood Pressure

**(DM2) **Type-2 Diabetes Mellitus

**(eNOS) **Endothelial Nitric Oxide Synthase

**(FAS) **Fatty Acid Synthase

**(FCV)**Fundacion Cardiovascular de Colombia

**(FFA) **Free Fatty Acids

**(FMD) **Flow-Mediated Dilation

**(GDPH) **Glycerol-2 Dehydrogenate Phosphate

**(HOMA) **The Homeostasis Model Assessment

**(IFG) **Impaired Fasting Glucose

**(IGT) **Impaired Glucose Tolerance

**(IL-6) **Interleukine 6

**(IRS-1 and IRS-2) **Insulin Receptor Substrate Type 1 and 2

**(MBP) **Mean Blood Pressure

**(MMP-2) **Matrix Metalloproteinase-2

**(NADPH) **Nicotinamide Adenine Dinucleotide Phosphate Hydrogen

**(NO) **Nitric Oxide

**(OGTT) **Oral Glucose Tolerance Test

**(PAI-1) **Plasminogen Activator Inhibitor-1

**(PKC) **Proteinkinase C

**(PPARs) **Peroxisome Proliferator-Activated Receptors

**(RAS) **Renin-Angiotensin-Aldosterone System

**(ROS) **Reactive Oxygen Species

**(SBP) **Systolic Blood Pressure

**(SD) **Standard Deviation

**(TLC) **Therapeutic Life-Style Change Program

**(TNF-α) **Tumor Necrosis factor alpha

**(tPA) **Tisular Plasminogen Activator

**(UPC-2) **Uncoupling Protein 2

**(W/H-R) **Waist-Hip Relation

## Competing interests

The authors declare that they have received a grant from AstraZeneca to develop the present project.

## Authors' contributions

PL-J who has made substantial contributions to the conception and design of the study, will be co-responsible for the overall administration and direction of the project, the analysis and interpretation of data and will give the final approval of the version to be published. VL participated in the design of the project and will be co-responsible for the overall administration and direction of the study. FS made substantial contributions to the conception and design of the study and was involved in the draft of the manuscript. LPP and CFR-C contributed to the design of the study, were involved in drafting the manuscript, will be responsible for the recruitment and the follow-up of the patients enrolled, and will also participate in the analysis and interpretation of data. GM contributed to the design of the study, will contribute to the acquisition of data, and will be responsible for overseeing the application of the protocol by monitoring the clinical trial to ensure compliance with the Good Clinical Practice guidelines. All authors read and approved the final manuscript.
